# Lipidomic Profiling of Hyperacute Ischemic Heart Disease and Toxic Deaths: A Forensic Investigation into Metabolic Biomarkers

**DOI:** 10.3390/ijms26189031

**Published:** 2025-09-17

**Authors:** Davide Radaelli, Monica Concato, Tommaso Bruscagin, Gianfranco Sinagra, Mariano Stornaiuolo, Stefano D’Errico

**Affiliations:** 1 Department of Medicine, Surgery and Health, University of Trieste, 34149 Trieste, Italy; monica.concato@phd.units.it (M.C.); tommaso.bruscagin@studenti.units.it (T.B.); gianfranco.sinagra@asugi.sanita.fvg.it (G.S.); sderrico@units.it (S.D.); 2 Department of Pharmacy, University of Naples Federico II, 80131 Naples, Italy; mariano.stornaiuolo@gmail.com

**Keywords:** sudden cardiac death, lipidomic, ischemic heart disease, forensic pathology, metabolic biomarkers

## Abstract

Sudden cardiac death (SCD) presents diagnostic challenges in distinguishing hyperacute ischemic heart disease (IHD) from drug-related fatalities. This pilot study leverages untargeted lipidomics to identify myocardial lipid biomarkers, analyzing heart tissue from six forensic cases (three hyperacute IHD and three drug deaths) via UHPLC-Q-TOF mass spectrometry. Data preprocessing (normalization, transformation, scaling) and multivariate analyses (PCA, PLS-DA) revealed distinct lipid profiles. Three lipids—PC 16:0_16:2, SM 34:1;3O, and PC O-40:5_C—were significantly upregulated in hyperacute IHD (FDR < 0.05), linked to glycerophospholipid metabolism and autophagy dysregulation. Machine learning models (SVM, random forest) achieved 66.7% accuracy in classifying etiology, with triacylglycerols and sphingomyelins as key discriminators. Toxic deaths showed elevated phosphatidylinositols (e.g., PI 38:4) and hexosylceramides. Despite the limited sample size, this work highlights lipidomic potential to complement traditional autopsies in SCD diagnostics. Findings implicate membrane remodeling and sphingolipid signaling in hyperacute IHD pathogenesis. Future studies with expanded cohorts are crucial to validate biomarkers and elucidate mechanisms.

## 1. Introduction

Sudden cardiac death (SCD) remains a significant challenge in forensic medicine, often presenting with inconclusive findings in conventional autopsies. Among these, “hyperacute” ischemic heart disease (IHD) represents rapid-onset myocardial ischemia leading to death, often challenging to diagnose post-mortem due to limited pathological signs, such as coronary arteries with significant but not complete occlusions. In these cases, the classification relies on the observer’s judgment of stenosis severity, which introduces potential bias and variability in diagnosis [1,2]. Similarly, toxic/drug-related deaths comprise fatalities related to drug intoxication with potential cardiac involvement, representing another diagnostically complex SCD subgroup. In recent years, metabolomics has emerged as a promising tool in forensic investigations, offering new insights into the biochemical state of an organism at the time of death [3].

Metabolomics, the youngest of the “omics” sciences, focuses on the comprehensive analysis of small molecule metabolites (typically < 1.5 kDa) in biological systems. It provides a functional readout of cellular state, offering unique insights into physiological and biochemical processes. There are two primary approaches in metabolomics research: targeted and untargeted [3]. Targeted metabolomics focuses on measuring specific, predefined sets of metabolites. It is hypothesis-driven and often used when researchers have prior knowledge of the metabolites of interest [4]. Untargeted metabolomics instead aims to measure as many metabolites as possible within a sample. For this reason, it is often used for hypothesis generation and discovery of novel metabolic pathways or biomarkers [5].

Its application in forensic science has expanded rapidly in recent years, with researchers exploring its potential in various areas such as post-mortem interval estimation, cause of death determination, and toxicological analyses [3,6,7]. Its power lies in detecting subtle changes in metabolic profiles that may not be apparent through traditional forensic methods.

Recent studies have explored the use of metabolomics in investigating SCD. In 2023 metabolomics and machine learning techniques have been applied in SCD cases. The authors analyzed blood and heart samples from SCD cases [8]. By integrating these metabolomic profiles with machine learning algorithms, they achieved remarkable accuracy in distinguishing SCD from non-SCD cases. The best-performing model, a stacking algorithm, demonstrated an impressive 92.31% accuracy [8]. Another significant contribution to the field came from a large cohort study published in 2023, which examined the relationship between specific sphingolipids and SCD risk in older adults. The study analyzed 4612 plasma samples, including 215 subjects classified as SCD by a cardiologist [9]. Intriguingly, the researchers found that higher plasma levels of ceramide-16 (Cer-16) and sphingomyelin-16 (SM-16) were associated with an increased risk of SCD. These associations remained robust even after adjusting for various risk factors, suggesting that these sphingolipids might play a more direct role in SCD pathogenesis than previously thought. Lastly, a 2024 manuscript revealed that SCD shares metabolic pathways with sudden cardiac arrest [10]. Common pathways included amino acid metabolism and lipid metabolism, highlighting the complex interplay of metabolic processes in cardiovascular events. The authors also identified SCD-specific alterations in starch/sucrose metabolism and nicotinate/nicotinamide metabolism pathways [10]. While these studies have provided valuable insights, it is crucial to recognize their limitations. In the first manuscript, the authors do not differentiate between specific causes of SCD, such as coronary artery disease, cardiomyopathies, or channelopathies, instead grouping them under the general SCD umbrella [8]. In the second one, the diagnosis of SCD was not autopsy-based, but the SCD individuals were selected by a cardiologist [9]. Notably, as the third study, the analyses were performed on plasma samples, which are not ideal for forensic matrices due to post-mortem changes [10,11].

To address these limitations and further explore the metabolic profiles associated with SCD, we conducted untargeted lipidomic analyses on fresh frozen heart tissue samples from six subjects. These individuals were either enrolled in the Friuli-Venezia Giulia Sudden Cardiac Death Registry in the Young or were cases from the routine medico-legal practice at the University of Trieste’s Department of Legal Medicine. This approach allows us to investigate SCD-associated metabolic profiles in a well-defined cohort, combining both registry data and forensic cases, while using a tissue type more suitable for post-mortem analysis.

## 2. Results

Lipidomic profiles were analyzed from six post-mortem samples: three from individuals who died from hyperacute ischemic heart disease (mean age 50 years) and three from individuals who died from toxic/drug abuse (mean age 34 ± 2 years).

The clinical and forensic characteristics of the subjects, including demographic data, coronary artery status, histological findings, pharmacological history, toxicological analyses, and final cause of death, are summarized in Table 1.

A total of 512 lipids were identified and quantified (Supplementary Materials Table S1). After quality filtering using a 25% SD threshold, 383 metabolites remained for further analysis. The data were subsequently normalized by mean, square root-transformed, and autoscaled prior to analysis.

### 2.1. Distinct Lipidomic Signatures Differentiate Hyperacute Ischemic Heart Disease from Toxic/Drug Abuse Deaths

**Figure 1:**
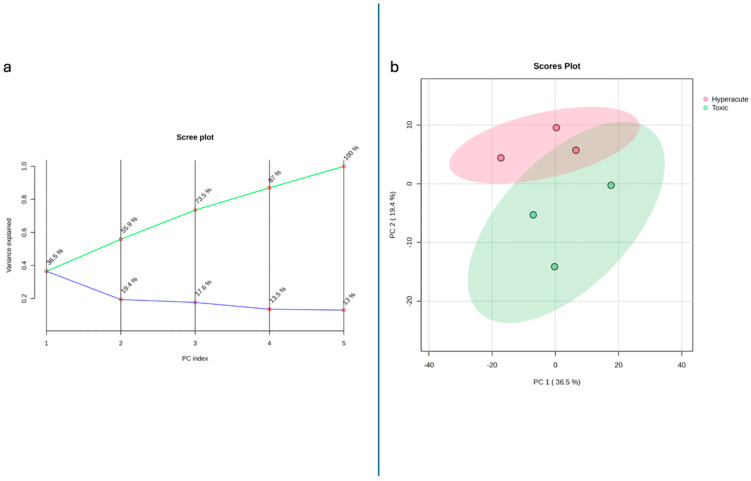
(**a**) Scree plot showing the different component contribution and (**b**) 2D scores plot.

Unsupervised principal component analysis (PCA) revealed distinct clustering patterns between the two groups (Figure 1a). The first three principal components (PCs) captured 73.5% of the total variance in the dataset, indicating that these components represent the major sources of variability in the lipid profiles. PC1, which accounted for the largest proportion of variance (36.5%), primarily separated hyperacute from toxic deaths. Hyperacute samples generally clustered in the upper quadrants of the scores plot with positive PC2 values (ranging from 5 to 10 on the PC2 axis), while toxic death samples displayed a broader distribution along PC2 (ranging from −15 to 0), primarily occupying the lower quadrants (Figure 1b). This suggests a greater degree of homogeneity in the lipid profiles of hyperacute deaths compared to toxic deaths. Along the PC1 axis, hyperacute samples were distributed between −20 and 0, whereas toxic samples showed a wider spread from −20 to 20, further supporting the observation of greater variability in the toxic death group. Examination of the loadings for PC1 revealed significant contributions from several lipid classes. Phosphatidylcholines exhibited strong positive loadings, particularly PC 16:0/16:0_A (0.081), PC O-36:1_A (0.082), and PC O-40:4_A (0.081), suggesting that these lipids are more abundant in hyperacute deaths. Diacylglycerols also contributed significantly to PC1, with DG 18:1/18:1 showing a positive loading (0.081). In contrast, PC O-28:0_A (−0.082), DG 16:1_16:1 (−0.082), and the phosphatidylinositol PI 18:0_20:4 (−0.082) displayed strong negative loadings, indicating their higher abundance in toxic deaths. The second principal component (PC2, 19.4% variance) revealed strong positive loadings from PC 15:0_20:4 (0.107), PC 16:0_18:0_A (0.104), and SM 20:1;2O/21:1_A (0.107). The ceramide HexCer 18:1;2O/24:0 (−0.096) and PC O-34:0_B (−0.096) showed notable negative contributions to PC2. The third component (PC3, 17.6% variance) displayed positive loadings from PC 14:0_16:0_B (0.095) and PC O-40:8_C (0.102), while PC O-36:0 (−0.118) and PS 18:0_18:1 (−0.118) contributed negatively to this component (Table 2).

### 2.2. Differential Lipid Abundance and Pathway Analysis

**Figure 2:**
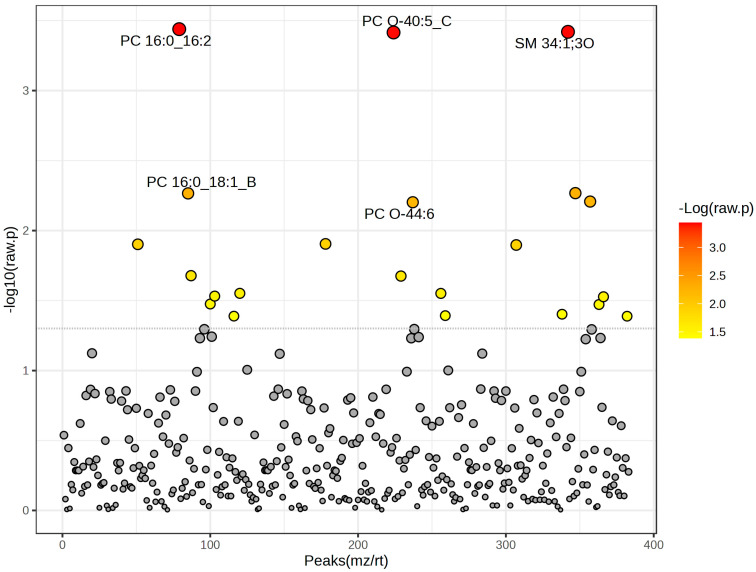
Raw *p*-values from *t*-test results. Dotted line refers to *p*-value threshold for significance.

To identify individual lipids that differed significantly in abundance between the two groups, a *t*-test was performed. Examination of the −log10 (raw *p*-value) distribution across all detected peaks (m/z/rt) revealed 22 lipids with high statistical significance (Figure 2). To control for multiple comparisons, the false discovery rate (FDR) was calculated using the Benjamini–Hochberg procedure. After FDR correction, these three lipids remained significant (FDR < 0.05): PC 16:0_16:2 (t = 11.182, FDR = 0.049095), sphingomyelin SM 34:1;3O (t = 11.056, FDR = 0.049095), and PC O-40:5_C (t = 11.026, FDR = 0.049095), indicating their consistent and robust differential abundance between hyperacute and toxic deaths. Several other lipid species showed moderate significance in raw *p*-values but did not meet the FDR threshold for significance, including SM 40:2;2O_A (t = 5.4796), PC 16:0_18:1_B (t = 5.4733), triacylglycerol TG 16:0_18:1_20:4 (t = 5.2722), and PC O-44:6 (t = 5.2559), all with FDR values of 0.34317 (Table 3).

**Figure 3:**
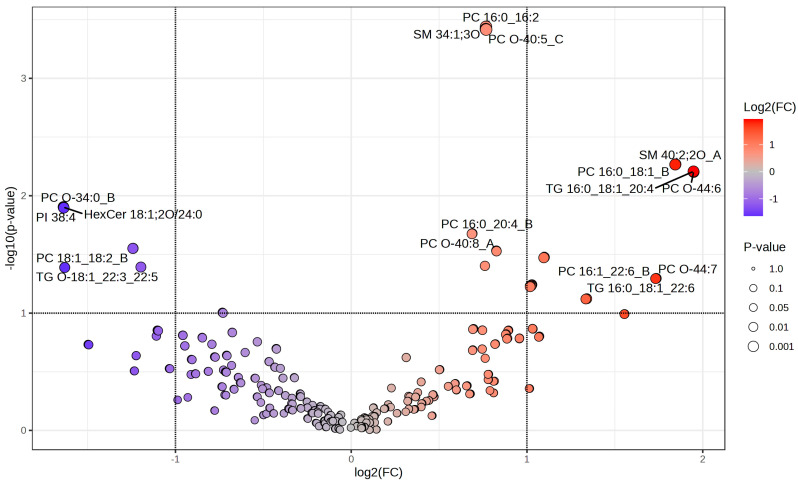
Volcano plot.

Volcano plot analysis revealed distinct changes in lipid profiles between hyperacute and toxic death samples (Figure 3 and Table 4). The distribution of lipid species showed both significant up- and down-regulation, with log2 fold changes ranging from −1.64 to 1.95 (Table 3). Three lipid species demonstrated high statistical significance (−log10 (*p*-value) > 3.4) and substantial fold changes: PC 16:0_16:2, SM 34:1;3O, and PC O-40:5_C. These lipids were all significantly upregulated in hyperacute deaths compared to toxic deaths. Several other lipids were notably upregulated in hyperacute compared to toxic deaths: PC O-44:6 (log2 (FC) = 1.95), TG 16:0_18:1_20:4 (log2 (FC) = 1.95), SM 40:2;2O_A (log2 (FC) = 1.84), and PC 16:0_18:1_B (log2 (FC) = 1.84). Conversely, significant downregulation was observed in PC O-34:0_B (log2 (FC) = −1.64), HexCer 18:1;2O/24:0 (log2 (FC) = −1.64), PI 38:4 (log2 (FC) = −1.64), and PC 18:1_18:2_B (log2 (FC) = −1.63) in hyperacute deaths compared to toxic deaths. The majority of detected lipids showed moderate changes, with log2 fold changes between −1 and 1, as visualized in the fold change distribution plot.

**Figure 4:**
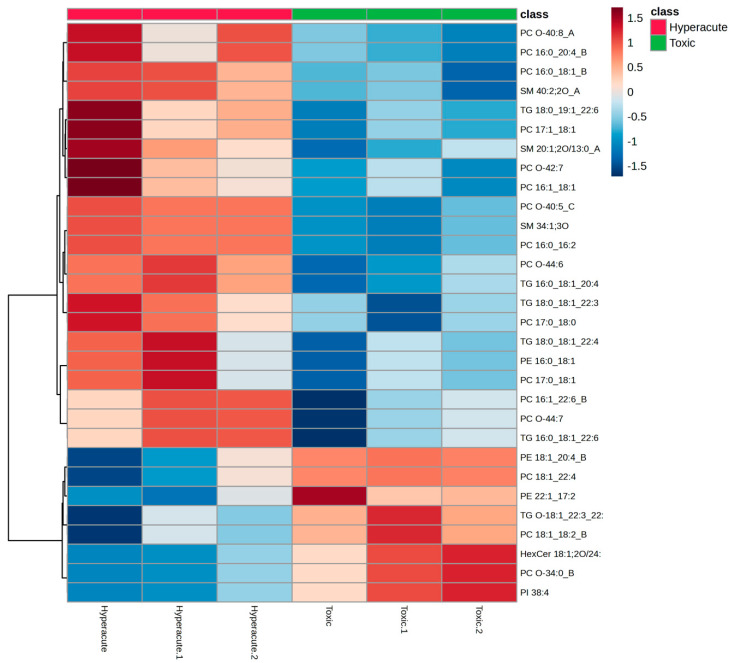
Top 25 metabolites clustered heatmap.

Hierarchical clustering analysis, which groups samples and lipids based on similarity in their abundance patterns, revealed distinct lipid signatures between hyperacute and toxic death samples (Figure 4). The heatmap visualization identified two main clusters with opposing abundance patterns. The upper cluster showed predominantly higher abundance in hyperacute samples, with PC O-40:8_A, PC 16:0_20:4_B, and PC 16:0_18:1_B displaying the strongest positive values (>1.0). Additional hyperacute-associated lipids included SM 40:2;2O_A and TG 18:0_19:1_22:6, showing moderate elevation (0.5–1.0). Conversely, the lower cluster demonstrated increased abundance in toxic samples, particularly PE 18:1_20:4_B, PC 18:1_22:4, and PE 22:1_17:2, with HexCer 18:1;2O/24:0, PC O-34:0_B, and PI 38:4 showing the strongest upregulation.

### 2.3. Potential Biomarkers for Hyperacute Ischemic Heart Disease and Toxic/Drug Abuse Deaths

**Figure 5:**
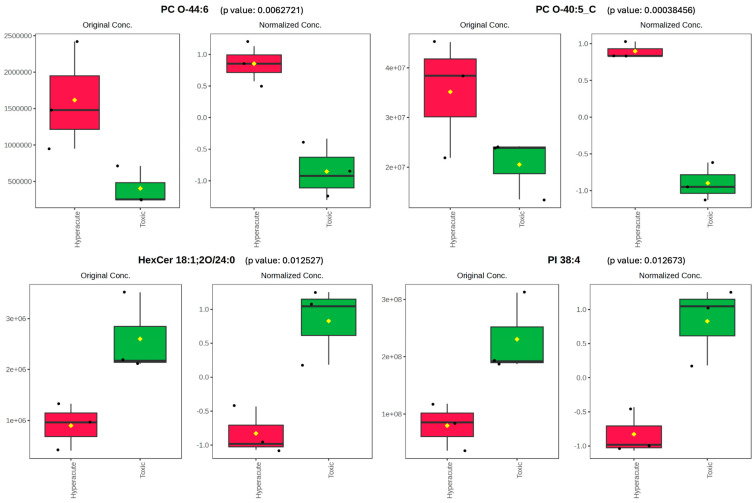
Original and normalized concentration of PCO-44:6, PC O-40:5 HexCer 18:2;2O/24:0 and PI38:4. Black dots represent the data, diamonds represent the mean between the data.

To further investigate the specific lipids driving the observed differences between the two groups, we examined the concentration patterns of key lipid species. Two phosphatidylcholines, PC O-40:5_C and PC O-44:6, showed significant elevation in hyperacute samples compared to toxic samples. PC O-40:5_C displayed a marked elevation in hyperacute samples (original peak intensity ~4.0 × 10^7^) compared to toxic samples (~2.0 × 10^7^), with normalized intensity showing clear separation (0.8 vs. −1.0). Similarly, PC O-44:6 exhibited higher levels in hyperacute cases (original intensity ~1.5 × 10^6^) versus toxic cases (~4.0 × 10^5^), with normalized values of 0.8 and −0.9, respectively. Conversely, PI 38:4 and HexCer 18:1;2O/24:0 demonstrated significantly higher intensity in toxic cases. PI 38:4 showed significantly higher intensity in toxic samples (original concentration ~2.5 × 10^8^) compared to hyperacute samples (~8.0 × 10^7^), with normalized values of 1.0 and −0.8, respectively. HexCer 18:1;2O/24:0 followed a similar pattern, with elevated levels in toxic samples (original intensity ~2.5 × 10^6^) versus hyperacute samples (~1.0 × 10^6^), showing normalized intensity of 0.8 and −0.8, respectively (Figure 5).

**Figure 6:**
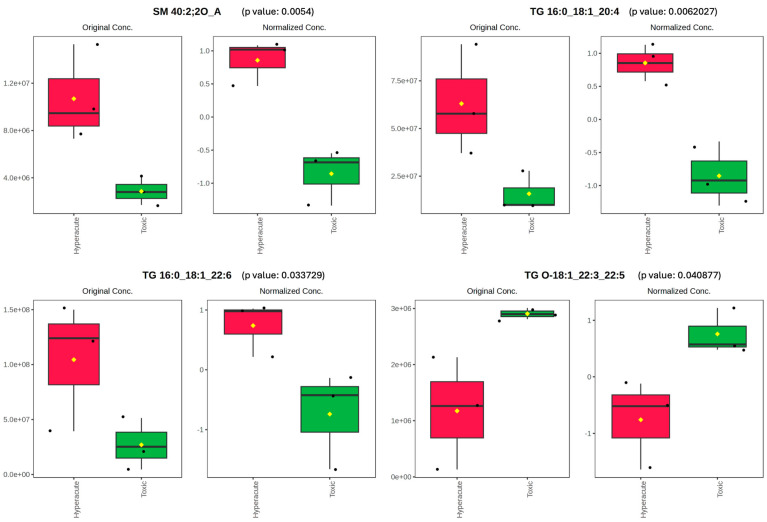
Original and normalized concentration of SM 40:2;2O, TG 16:0_18:1_20:4, TG 16:0_18:1_22:6 and TG O-18:1_22:3_22:5. Black dots represent the data, diamonds represent the mean between the data.

Sphingomyelin and triacylglycerol species also revealed distinct concentration patterns between the two groups. SM 40:2;2O_A showed marked elevation in hyperacute samples (original concentration ~1.2 × 10^7^) compared to toxic samples (~3.0 × 10^6^), with normalized concentrations showing clear separation (1.0 vs. −0.8). The box plots demonstrated minimal overlap between groups, with consistent median values and relatively low within-group variability. Triacylglycerols exhibited varying patterns of distribution. TG 16:0_18:1_20:4 and TG 16:0_18:1_22:6 showed significant elevation in hyperacute cases, with original concentrations of ~7.5 × 10^7^ and ~1.2 × 10^8^, respectively, compared to toxic cases (~1.5 × 10^7^ and ~2.5 × 10^7^). Their normalized values demonstrated consistent separation between groups (0.8 to 1.0 for hyperacute vs. −0.9 to −0.8 for toxic). Notably, TG 16:0_18:1_22:6 displayed the largest absolute concentration difference between groups. Conversely, TG O-18:1_22:3_22:5 showed an opposite trend with higher concentrations in toxic samples (original concentration ~3.0 × 10^6^) versus hyperacute samples (~1.2 × 10^6^), with normalized values of 0.8 and −0.8, respectively. The box plots (Figure 6) demonstrate minimal overlap between groups for all four lipid species, with consistent patterns of elevation specific to each death type. The median values showed clear separation, and the interquartile ranges indicated relatively low variability within each group, suggesting these lipids as potential biomarkers for distinguishing between hyperacute and toxic death cases.

### 2.4. Multivariate Statistical Analyses

**Figure 7:**
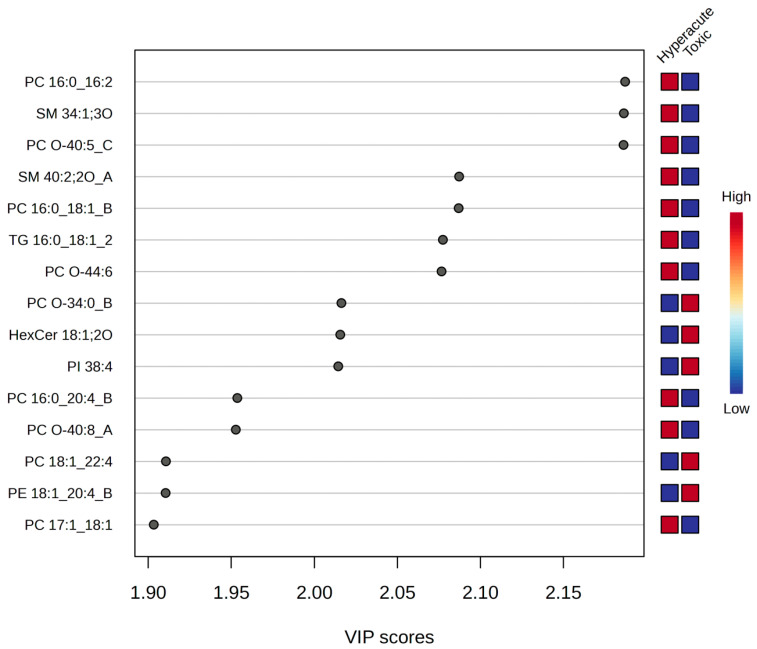
PLS-DA VIP score.

To further explore the discriminatory power of the lipidomic data, we employed multivariate statistical analyses. Orthogonal partial least squares discriminant analysis (OPLS-DA) and partial least squares discriminant analysis (PLS-DA) models were constructed to maximize the separation between hyperacute and toxic death samples based on their lipid profiles. Both models revealed distinct lipid signatures associated with each group (Figure 7). Variable Importance in Projection (VIP) analysis, which ranks variables based on their contribution to the model’s discriminatory power, identified fifteen lipids with significant discriminatory power (VIP > 1.90). Three phosphatidylcholines (PC 16:0_16:2, PC O-40:5_C, and PC 16:0_18:1_B) and two sphingomyelins (SM 34:1;3O and SM 40:2;2O_A) showed the highest VIP scores (2.15–2.10) and were consistently elevated in hyperacute deaths. Conversely, PC O-34:0_B, HexCer 18:1;2O, and PI 38:4 demonstrated strong association with toxic deaths (VIP = 2.00).

**Figure 8:**
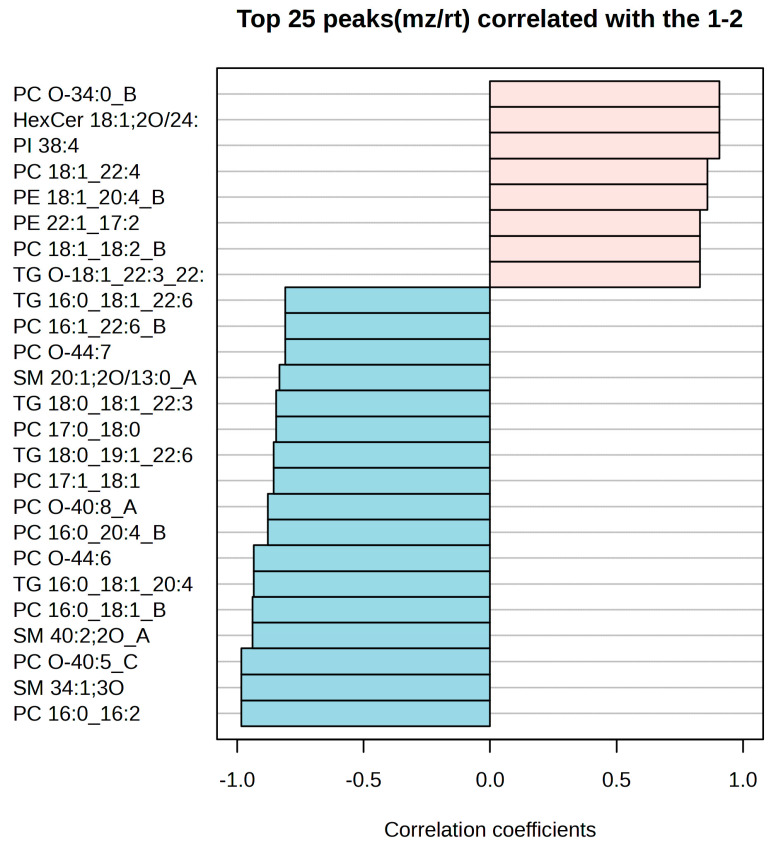
Correlation coefficients showing metabolite and PC correlations. Blue bars mean negative correlation, while red bars mean positive correlation.

Correlation analysis among these VIP lipids identified two distinct clusters with opposing patterns (Figure 8). The toxic death-associated cluster showed strong positive correlations (r > 0.80) among PC O-34:0_B, HexCer 18:1;2O/24:0, PI 38:4, PC 18:1_22:4, and PE 18:1_20:4_B, indicating that these lipids tend to change in the same direction in toxic deaths. The hyperacute death-associated cluster displayed strong negative correlations (r < −0.80) and included PC 16:0_16:2, SM 34:1;3O, PC O-40:5_C, and PC 16:0_18:1_B, suggesting that these lipids tend to change in the opposite direction to the toxic death-associated lipids in hyperacute deaths. Both OPLS-DA and PLS-DA models demonstrated consistent lipid importance patterns, with VIP scores above 1.90 indicating robust discriminatory power for the identified lipids.

### 2.5. Empirical Bayes, Significance Analysis of Microarrays and Kmeans

**Figure 9:**
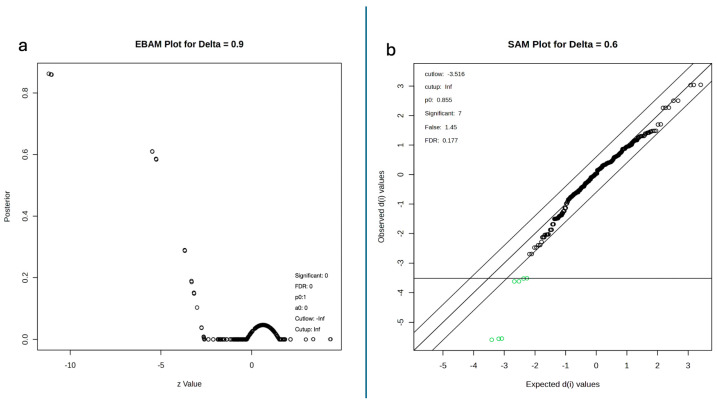
(**a**) EBAM plot results showing no statistically significant lipids. (**b**) SAM plot.

To further assess the statistical significance of the observed lipid changes, we employed empirical Bayes analysis (EBAM) and significance analysis of microarrays (SAM). The EBAM plot (delta = 0.9) showed that none of the lipids met the criteria for statistical significance in terms of differential abundance between the two causes of death; however, posterior probability distribution shows three features having high posterior probabilities (>0.8) at the extreme negative z-values (−10), indicating strong differential expression between the two groups (Figure 9a). The SAM analysis (delta = 0.6) identified seven significant features (FDR = 0.177, p0 = 0.855), with observed d (i) values ranging from −3.5 to 3.0, further supporting the presence of differentially abundant lipids between hyperacute and toxic deaths (Figure 9b).

**Figure 10:**
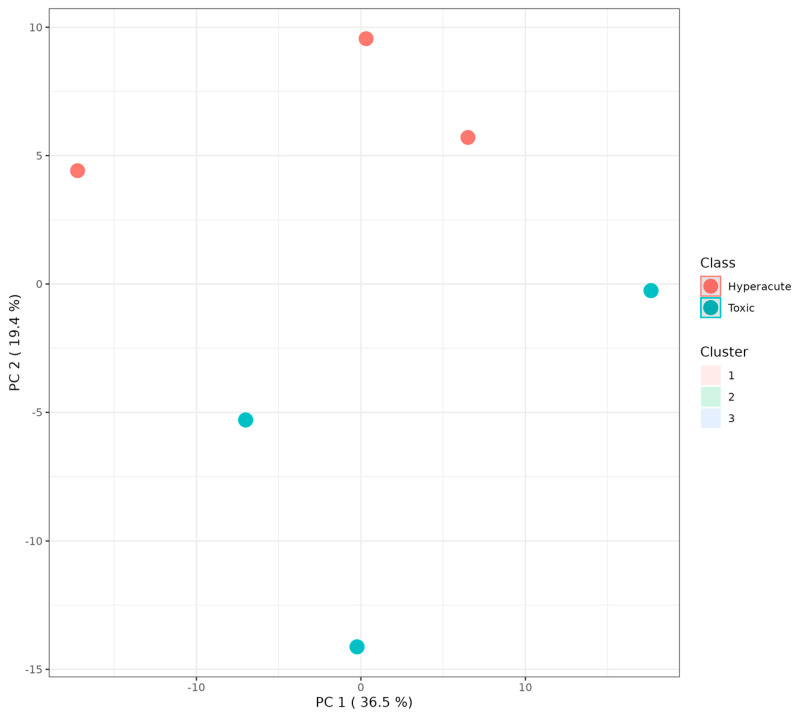
K-means principal component analyses showing the division into three clusters. Red dots represent hyperacute data, light-blue dots represents the toxic data.

K-means clustering analysis was performed on the principal components to identify potential subgroups within each cause of death. The analysis demonstrated clear separation between hyperacute and toxic death samples (Figure 10). The hyperacute samples clustered in three distinct positions along PC1 (36.5% variance) and PC2 (19.4% variance): one group at (−10, 5), another at (0, 10), and a third at (10, 5). In contrast, toxic samples formed two distinct clusters: one at (−10, −5) and another at (0, −15), showing clear separation from hyperacute samples, particularly along PC2. This clustering pattern suggests three distinct lipid profiles within hyperacute deaths and two within toxic deaths, indicating potential subgroups within each death type that may warrant further investigation.

### 2.6. Machine Learning and Clustering Analyses

**Figure 11:**
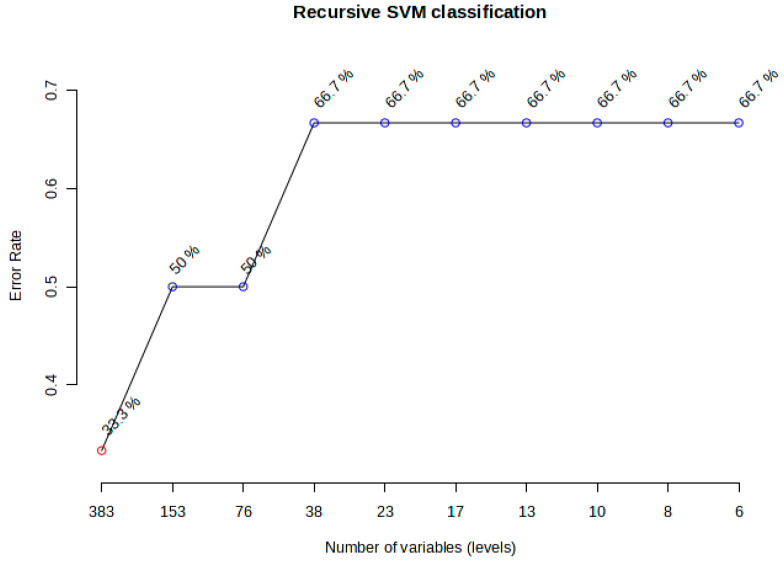
SVM analyses showing no significant accuracy changes from 38 to 6 variables. Blue dots represent the accuracy levels reached at different levels.

To further assess the ability of lipidomic profiles to distinguish between hyperacute ischemic heart disease and toxic/drug abuse deaths, we employed machine learning approaches. Recursive support vector machine (SVM) classification was performed with repeated cross-validation to identify an optimal set of discriminatory lipids (Figure 11). The model demonstrated optimal performance with 38 variables, achieving 66.7% accuracy in classifying samples into their respective death categories. Notably, this accuracy remained stable through further feature reduction down to six variables, suggesting a core set of highly discriminatory lipids.

**Figure 12:**
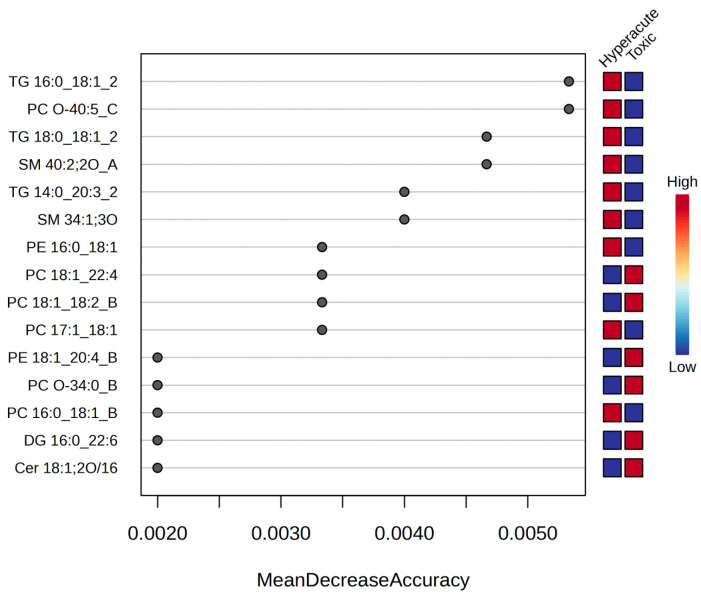
Random forest mean decrease accuracy.

Random forest analysis was employed to identify key lipids contributing to the classification. The variable importance plot revealed three distinct tiers of discriminating lipids based on mean decrease accuracy. TG 16:0_18:1_2, PC O-40:5_C, TG 18:0_18:1_2, and SM 40:2;2O_A have the highest mean decrease accuracy, indicating they are the most important lipids for distinguishing between the two groups (Figure 12).

**Figure 13:**
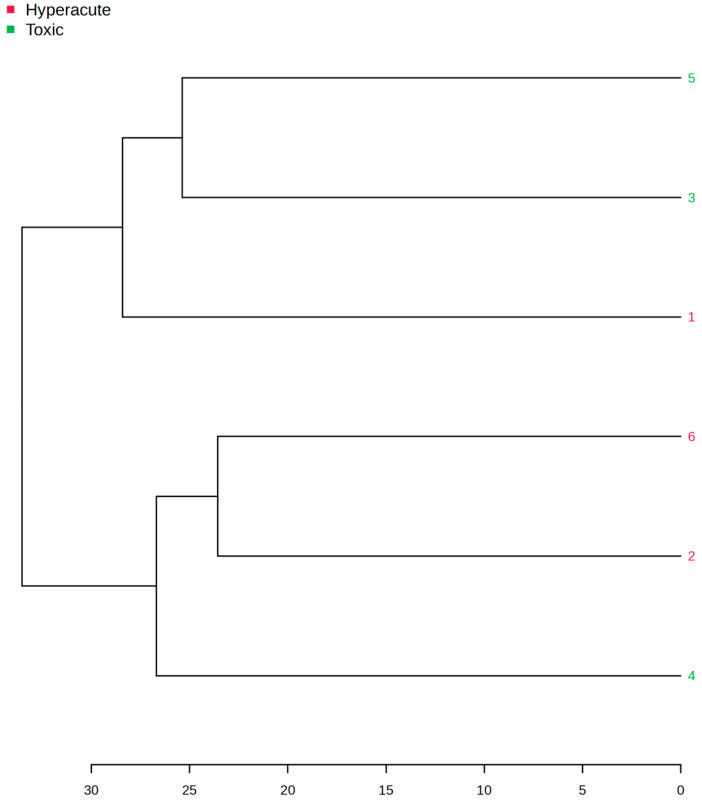
Tree clustering showing the distinct groups.

Hierarchical clustering analysis of the samples based on the identified discriminatory lipids revealed distinct sample groupings, with hyperacute and toxic samples forming separate clusters at a height of approximately 25 units. In the hyperacute group samples 1 and 6 are more closely related to each other than to sample 2, indicating that samples 1 and 6 have more similar lipid profiles. Sample 2 appears to have a slightly more distinct lipid profile within the hyperacute group. Similarly, in the toxic group, instead, samples 3 and 5 cluster closely together, while sample 4 still clusters with the other toxic samples but is a bit more distant, indicating some differences in its lipid composition (Figure 13).

### 2.7. Pathway Enrichment Analysis

To further understand the biological processes underlying the observed lipidomic changes, we conducted pathway enrichment analysis. Several pathways were significantly altered (Benjamini-corrected *p* < 0.05) between hyperacute and toxic death samples. Several pathways were significantly altered (Benjamini-corrected *p* < 0.05) between hyperacute and toxic death samples. The analysis of lipid metabolic pathways revealed significant alterations across multiple biological processes. Most notably, the Glycosylphosphatidylinositol (GPI)-anchor biosynthesis pathway demonstrated substantial modifications, with 28.57% of its lipids being converted (*p* = 0.000435, Benjamini correction = 0.003480). Glycerophospholipid metabolism exhibited the highest conversion rate, with 42.86% of its lipids modified, including key molecules C00157, C00350, and C01194 (*p* = 0.003109, Benjamini correction = 0.012438). Interestingly, both autophagy-related pathways showed significant alterations, with autophagy-other and autophagy-animal displaying identical conversion rates of 28.57% (*p* = 0.000435 and *p* = 0.000864, respectively). The Retrograde endocannabinoid signaling pathway also demonstrated notable changes with a 28.57% conversion rate (*p* = 0.003935) (Table 5).

On the contrary, the hyperacute altered pathways analyses revealed significant alterations in four distinct pathways (*p* < 0.05; Table 6). The necroptosis pathway showed the most significant changes (*p* = 0.022), followed by choline metabolism in cancer (*p* = 0.028), Retrograde endocannabinoid signaling (*p* = 0.044), and the sphingolipid signaling pathway (*p* = 0.050). Each pathway exhibited a consistent conversion rate of 33.33% of their constituent lipids. Notably, C00157 (3-sn-phosphatidylcholine) was the most frequently converted lipid, appearing in multiple pathways including choline metabolism and Retrograde endocannabinoid signaling, while C00550 (sphingomyelin) was predominantly involved in sphingolipid-related pathways. After applying multiple testing corrections, these associations remained noteworthy under Benjamini correction (0.124), though they did not meet the more stringent Bonferroni-corrected threshold. The larger pathways, such as arachidonic acid metabolism with 75 lipids, showed less significant changes (*p* = 0.364), suggesting that lipid modifications may be more impactful in smaller, specialized metabolic pathways.

These results suggest that membrane lipid remodeling, particularly involving glycerophospholipids, and autophagy–necrophagy-related processes play crucial roles in distinguishing between hyperacute ischemic heart disease and toxic/drug abuse deaths.

## 3. Discussion

The present pilot study explores a new approach in forensic lipidomics by seeking to identify unique lipid profiles associated with specific causes of sudden unexpected death. To our knowledge, this is the first study to correlate lipidomic analyses with a definitive cause of death determined through extensive forensic investigation. By focusing on differentiating SCD due to hyperacute ischemic heart disease from death due to drug abuse, we sought to uncover potential lipid biomarkers that could aid in forensic investigations and shed light on the underlying pathophysiology of these conditions.

Our findings not only corroborate existing knowledge of lipid alterations in cardiac ischemia but also provide a more nuanced understanding of the lipidomic landscape in hyperacute IHD. Three key lipid species with strong discriminatory power have been found (PC 16:0_16:2, SM 34:1;3O, and PC O-40:5_C), all significantly upregulated in hyperacute deaths. The elevation of these specific phosphatidylcholine and sphingomyelin species suggests substantial membrane remodeling during acute cardiac ischemia. A comprehensive review of 16 cohort studies found that phosphatidylcholines containing saturated and monounsaturated fatty acyl chains were positively associated with cardiovascular disease outcomes, aligning with our observation of elevated PC 16:0_16:2 and PC O-40:5_C in hyperacute deaths [12]. The biological significance of elevated phosphatidylcholines in hyperacute cardiac death can be explained through several mechanisms. First, PCs are especially prone to oxidation during ischemic events, leading to the formation of oxidized phosphatidylcholines (OxPCs) [13]. These oxidized species, particularly those containing fragmented fatty acids, are elevated in STEMI patients and contribute to increased infarction size and ventricular remodeling. Second, PC alterations affect membrane fluidity and permeability, potentially disrupting cellular calcium homeostasis during acute cardiac events [14].

The significant elevation of sphingomyelin species, particularly SM 40:2;2O_A, in hyperacute ischemic heart disease cases provides crucial insights into the role of sphingolipids in acute cardiac events. This finding aligns with and extends beyond the Cardiovascular Health Study, which demonstrated that sphingolipids containing palmitic acid were associated with a 37% increased risk of sudden cardiac death [9]. While previous studies focused on plasma samples, our tissue-based analysis reveals parallel sphingolipid alterations in cardiac tissue, suggesting these lipids play fundamental roles in cardiac death mechanisms across different biological compartments. Biologically elevated sphingomyelins in hyperacute cardiac death can be explained through several mechanisms. Sphingolipids, in fact, are not merely structural components of cell membranes but act as bioactive signaling molecules that regulate crucial cellular processes, including inflammation, oxidative stress, and cell death pathways [15]. Recent studies have shown that sphingolipids contribute to atherosclerotic plaque inflammation and instability through multiple pathways, including macrophage activation and foam cell formation [16]. The elevation of specific sphingomyelin species, then, suggests active membrane remodeling during acute cardiac events. This is particularly relevant as sphingomyelins can account for 70–80% of all phospholipids in atherosclerotic necrotic cores [16]. Their accumulation may reflect both ongoing tissue damage and attempted cellular repair mechanisms. Furthermore, sphingomyelin species have been shown to regulate calcium homeostasis and cellular stress responses, both critical factors in acute cardiac injury [17]. The correlation between sphingolipid levels and cardiac injury markers suggests their potential role in disease progression. Recent studies have demonstrated that sphingolipids can serve as both biomarkers and active mediators of cardiovascular pathology [18]. Studies have shown that ceramides accumulate rapidly during cardiac ischemia–reperfusion injury, particularly those containing palmitic acid, stearic acid, and arachidonic acid [19]. This accumulation leads to mitochondrial dysfunction, enhanced ROS formation, and initiation of cytochrome C release, promoting apoptosis through caspase 3 pathways [20]. Their elevation in tissue samples may precede traditional markers of cardiac damage, offering potential early diagnostic value. The pathophysiological implications of elevated sphingomyelins extend beyond their role as structural membrane components. These lipids are now recognized as crucial modulators of inflammatory pathways involved in cardiovascular disease progression. They can regulate the expression of pro-inflammatory mediators and activate signaling pathways involved in immune cell recruitment and activation. This inflammatory component is particularly relevant in the context of acute cardiac events, where rapid immune responses can significantly impact tissue damage and patient outcomes [18]. Moreover, the specific elevation of SM 40:2;2O_A in our hyperacute cases may represent a novel tissue-specific marker of cardiac injury. This finding is particularly significant as it provides direct evidence of sphingolipid alterations in cardiac tissue during acute events, complementing previous plasma-based studies. The consistency between tissue and plasma findings suggests these lipid alterations reflect fundamental pathophysiological processes rather than localized changes. The altered phospholipid profiles we observed likely reflect both active membrane remodeling and cellular stress responses. During ischemia, the activation of calcium-dependent lipases and phospholipases contributes to membrane disruption [17]. In general, the phospholipid accumulation process is particularly relevant, where phospholipids serve as key mediators in several pathways as signal transduction during acute cardiac stress (as regulation of inflammatory responses, modulation of cell death mechanisms and calcium homeostasis), and their dysregulation can lead to membrane instability and dangerous ventricular arrhythmias [13].

Also, elevation of triacylglycerol species in hyperacute cardiac deaths provides important insights into altered energy metabolism and lipotoxicity during acute cardiac events. Our analysis, indeed, identified distinct TAG signatures, particularly TG 16:0_18:1_20:4 and TG 16:0_18:1_22:6, which were significantly elevated in hyperacute compared to toxic deaths. These findings align with recent lipidomic profiling studies showing that specific TG species serve as strong predictors of cardiovascular events [21]. The biological significance of elevated TGs in hyperacute cardiac death can be explained through several mechanisms. First, the accumulation of specific TG species, particularly those containing long-chain fatty acids (such as TG 16:0_18:1_20:4), suggests impaired fatty acid oxidation during acute ischemia. This metabolic disruption likely reflects the heart’s unsuccessful attempt to switch from fatty acid to glucose metabolism under ischemic conditions [22]. The presence of polyunsaturated fatty acids in these TG species (20:4 and 22:6) is particularly noteworthy, as these lipids are known to influence inflammatory responses and membrane stability. Second, the elevation of TGs containing arachidonic acid (20:4) suggests increased inflammatory signaling, as arachidonic acid serves as a precursor for various inflammatory mediators. This aligns with recent findings showing that TG-rich lipoproteins contribute to cardiovascular risk through both direct and indirect inflammatory mechanisms [22]. Third, the accumulation of docosahexaenoic acid-containing TGs (22:6) may represent a failed cardioprotective mechanism, as DHA is known to have anti-inflammatory properties. The elevation of TG 16:0_18:1_22:6 could indicate the heart’s attempt to sequester protective fatty acids during acute stress. Recent epidemiological studies have demonstrated that TG-containing lipoproteins, including remnant particles, show a clear relationship with cardiovascular disease risk [22]. Our findings extend this understanding to the tissue level, suggesting that specific TG species might serve not only as biomarkers but also as indicators of the underlying pathophysiological processes in acute cardiac death. These alterations in TG profiles may represent both maladaptive responses to acute cardiac stress and potential therapeutic targets. The identification of specific TG species associated with hyperacute cardiac death could lead to improved risk stratification and novel therapeutic strategies focusing on lipid metabolism.

The interpretation of lipid alterations in the toxic death group presents a complex scenario, because these lipid species can exhibit either elevation under toxic conditions or depletion in acute ischemia. However, it is known that lipids like ceramides accumulate in cardiac lipotoxicity [23], while some phosphatidylcholines and phosphatidylinositol showed a significant depletion [21]. So a true drug effect cannot be ruled out, especially because two subjects tested positive for cocaine, which has a known cardiac effect. In general, this group is characterized by elevated levels of specific lipid species, such as HexCer 18:1;2O/24:0, but also PC O-34:0_B and PI 38:4. These alterations suggest potential disruptions in membrane composition and signaling pathways induced by drug toxicity, highlighting the effect that toxic substances could have on cellular lipid metabolism. Furthermore, the broader distribution observed in the PCA plot for the toxic group indicates greater variability in their lipid profiles compared to the hyperacute IHD group. This variability likely reflects the diverse effects of different toxic agents on lipid metabolism and underscores the complexity of toxic-related deaths. The K-means clustering analysis further emphasized this heterogeneity, revealing two distinct subgroups within the toxic death samples, suggesting varying metabolic responses to different toxic substances.

The pathway analysis revealed fundamentally different metabolic signatures between hyperacute ischemic and toxic deaths, suggesting distinct mechanisms of cellular injury and death. In toxic deaths, the significant alterations in GPI-anchor biosynthesis (28.57% conversion) indicate substantial disruption of membrane protein organization. GPI anchors are crucial for tethering specific proteins to the plasma membrane and play vital roles in cellular signaling, adhesion, and immune responses [24]. The dysregulation of this pathway suggests compromised membrane protein localization and cellular signaling, potentially affecting cell survival mechanisms.

In hyperacute deaths, the prominence of necroptosis pathway alterations reveals active programmed cell death mechanisms. Recent evidence demonstrates that necroptosis plays a crucial role in myocardial homeostasis and ischemic injury through several mechanisms [25]. During acute cardiac events, key mediators of necroptosis, including RIPK1, RIPK3, and MLKL, are significantly upregulated, leading to disruption of plasma membrane integrity. This pathway is particularly relevant as necroptosis, unlike apoptosis, is a lytic form of cell death that releases pro-inflammatory intracellular contents, explaining the inflammatory component often observed in acute cardiac deaths. These distinct pathway alterations between toxic and hyperacute deaths not only reflect different mechanisms of cellular injury but also suggest potential therapeutic targets. While toxic deaths appear to primarily affect membrane organization and cellular energetics, hyperacute deaths show prominent activation of programmed cell death pathways and inflammatory signaling.

Several lipid species demonstrated strong potential as biomarkers. In hyperacute cases, PC O-40:5_C and PC O-44:6 showed consistent elevation, while SM 40:2;2O_A demonstrated marked elevation. For toxic cases, HexCer 18:1;2O/24:0 and PI 38:4 showed significant elevation. These distinct profiles could serve as diagnostic markers in forensic settings.

Our study presents several significant methodological strengths that enhance the reliability and applicability of our findings. The use of fresh frozen heart tissue samples provides direct insight into the cardiac tissue metabolic state at the time of death, offering more reliable metabolic profiles compared to plasma samples. This approach is particularly valuable in forensic settings, where post-mortem changes can significantly affect blood-based analyses. The clear differentiation between hyperacute ischemic deaths and toxic deaths, supported by comprehensive autopsy findings, allows for more precise metabolic profiling of specific death mechanisms, avoiding the limitations of previous studies that grouped different causes under the general SCD umbrella. Furthermore, our untargeted lipidomic approach using high-resolution mass spectrometry enabled the identification and quantification of 512 lipid species, providing unprecedented detail in the metabolic signatures of cardiac death.

However, several important limitations must be considered when interpreting our results. The limited sample size stems from the inherent challenges of forensic research methodology. Samples must be collected during autopsy procedures, while the definitive cause of death can only be established following comprehensive post-mortem investigations, creating an unavoidable bottleneck in sample collection. Another significant challenge lies in the fundamental nature of forensic research: the impossibility of establishing true control groups, as all samples inherently come from deceased individuals with various pathological conditions. To address this limitation, we utilized the toxic death group as our reference group, selected based on two critical factors. First, these cases represented the second most frequent cause of death in our cohort. Second, their primary mechanism of death involved central respiratory depression rather than direct cardiac failure. It is important to acknowledge that toxic conditions may cause cardiac alterations either directly or indirectly, through mechanisms distinct from those underlying ischemic heart disease. This factor introduces complexity in interpreting our findings. Notably, several cases in the toxic death group tested positive for drugs known to affect cardiac metabolism, such as cocaine and methadone [26,27]. These substances can induce metabolic disruptions, including lipid dysregulation, oxidative stress, and inflammation, which could contribute to the altered lipidomic profiles observed. Consequently, some of the lipid changes detected in this group may reflect direct pharmacological effects or secondary responses to drug-induced cardiac injury. Distinguishing drug-specific metabolic signatures from those associated with the cause of death remains a significant and challenging issue in forensic metabolomics.

Also, the significant age difference between groups (mean age 50 years for hyperacute vs. 34 years for toxic deaths) introduces a potential confounding factor, as age-related changes in lipid metabolism might influence the observed differences.

While matching for age was not possible due to real-world forensic limitations, future studies will be designed to include age-matched cohorts and/or perform statistical adjustment for age as a covariate in multivariate models.

Despite using fresh frozen tissue, some post-mortem changes in lipid profiles cannot be completely ruled out, and the time between death and sample collection could affect metabolite stability.

These limitations suggest several crucial directions for future research. First, validation studies in larger cohorts with matched age groups are essential to confirm our findings and establish more robust biomarker profiles. Second, longitudinal studies investigating the temporal dynamics of lipid changes during acute cardiac events could provide valuable insights into the progression of metabolic alterations leading to death. This could be particularly valuable for identifying early warning signs and potential therapeutic interventions. Third, the development of targeted assays for the most promising biomarker candidates (such as PC O-40:5_C and SM 40:2;2O_A for hyperacute deaths) could lead to practical diagnostic tools for forensic applications. Fourth, mechanistic studies exploring the biological pathways identified in our analysis, particularly the role of necroptosis in hyperacute deaths and glycerophospholipid metabolism in toxic deaths, could reveal new therapeutic targets. Additionally, future studies should explore the correlation between tissue and plasma lipid profiles, potentially leading to the development of less invasive diagnostic tools. The integration of lipidomic data with other molecular markers and clinical parameters could enhance our understanding of the pathophysiological mechanisms underlying different types of cardiac death. Finally, the potential application of these findings in ante-mortem risk stratification warrants investigation, as identifying high-risk individuals could lead to preventive interventions. These future directions aim not only to validate and extend our current findings but also to translate them into practical applications in both forensic and clinical settings.

## 4. Materials and Methods

### 4.1. Human Sample Collection During Forensic Autopsy

The regional register of sudden cardiac death and the related research were approved by the Friuli-Venezia Giulia Region Ethics Committee (Prot.N.0034183/PGEN/ARCS). The forensic autopsies were performed by the University of Trieste’s Department of Legal Medicine, Italy. A total of 6 individuals were enrolled in the study. The cardiac samples were collected during the autopsy after eviscerating the heart and before submerging it into formalin solution for subsequent analyses. To not alter the shape of the heart, a 6 mm dermopunch has been used for the purpose. Samples were collected from the anterior, lateral and posterior midventricular left and right ventricle walls. After the collection, samples were inserted into 2 ml micro vials and frozen with liquid nitrogen, then stored at −80° till the analyses. The causes of death were determined according to the SCD Friuli-Venezia Giulia Register Protocol. Three subjects showed critical coronary artery disease (stenosis >75), with unremarkable gross and microscopic examination; the control group included three other individuals who died from drug intoxication. The details are shown in Table 1.

### 4.2. Reagents

Chemicals were LC–MS-grade water (H_2_O), acetonitrile (ACN), methanol (CH_3_OH), isopropanol (IPA), 1-butanol (BuOH) and dichloromethane (CH_2_Cl_2_), all purchased from Merck, (Darmstadt, Germany).

### 4.3. Instrumentation

Lipidomics analysis was performed by RP-UHPLC on a Thermo Ultimate UHPLC system (Thermo Scientific, Bremen, Germany) coupled online to a TimsTOF Pro Quadrupole Time of Flight (Q-TOF) (Bruker Daltonics, Bremen, Germany) equipped with an Apollo II electrospray ionization (ESI) probe.

### 4.4. Sample Preparation

Frozen heart tissue samples were mechanically homogenized on ice using a bead mill homogenizer. Approximately 20 mg of tissue was processed per sample. The homogenized tissue was mixed with ice-cold dichloromethane (CH_2_Cl_2_) and vortexed vigorously. Samples were then centrifuged at 14,680 rpm for 10 min at 4 °C to separate phases. The resulting organic phase was collected and evaporated to dryness using a SpeedVac (Savant, Thermo Scientific, Milan, Italy). The dried lipid extracts were reconstituted in 100 μL of butanol/isopropanol/water (8:23:69 *v*/*v*/%) prior to analysis by UHPLC-QTOF.

### 4.5. Data Processing and Statistical Analyses

The data were analyzed using the web-based platform MetaboAnalyst 6.0. Before the analyses, the data were processed according to lipidomic standards and MetaboAnalyst’s recommended workflow for untargeted metabolomics data [28,29]. The processing steps were as follows:

1. Data filtering: A 25% filter based on standard deviation was applied to remove variables with low variation across samples, which are less likely to be informative; a total of 128 lipids were removed;
2. Normalization: Data were normalized by mean to correct for systematic differences between samples;
3. Data transformation: A square root transformation was applied to reduce the impact of heteroscedasticity and to make the data distribution more symmetric;
4. Scaling: Data were autoscaled (mean-centered and divided by the standard deviation of each variable) to make features more comparable.

**Figure 14:**
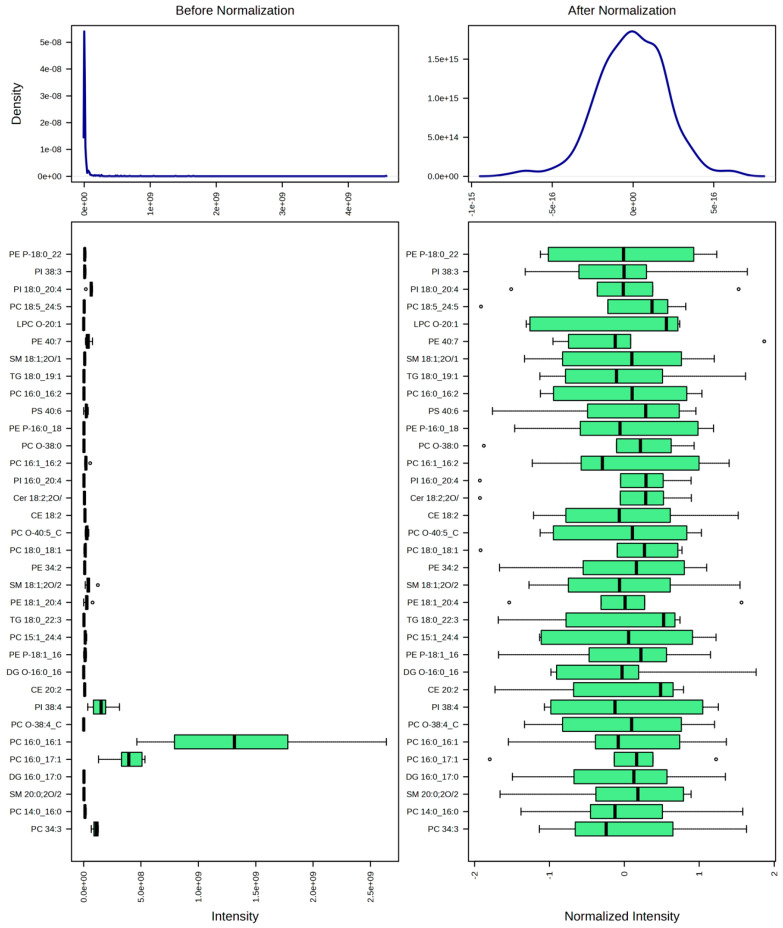
Data normalization showing a bell-shaped distribution.

The result of the data normalization process is shown in Figure 14.

After preprocessing, the data were ready for subsequent statistical and functional analyses within the MetaboAnalyst platform. One feature with a constant or single value across samples was found and deleted.

We employed multiple MetaboAnalyst modules for comprehensive statistical evaluation and data visualization: fold change analysis and univariate *t*-tests identified individual lipids with differential abundance between groups. Volcano plots combine fold changes and *p*-values for intuitive visualization of significant features. Pattern Hunter was utilized to explore specific correlational patterns among metabolites.

Multivariate analyses included Principal Component Analysis (PCA) performed on normalized and scaled data, extracting the first five principal components to investigate sample variance and clustering. Both hierarchical clustering and dendrogram visualization were performed using Euclidean distance and Ward’s linkage method to explore similarities among samples and metabolites. K-means clustering further subdivided samples into potential subgroups.

For predictive modeling, supervised machine learning algorithms were applied, including Support Vector Machine (SVM) with recursive feature elimination and Random Forest classification, to identify lipids with strong discriminatory power between hyperacute ischemic and toxic death groups. Models were evaluated by cross-validation to assess classification accuracy.

The metabolites showing significant differences between hyperacute and toxic death samples were analyzed using the LIPEA (Lipid Pathway Enrichment Analysis—https://hyperlipea.org/home, accessed on 22 October 2024) web-based tool, selecting Homo sapiens as the reference background [30].

## 5. Conclusions

This pilot study demonstrates the potential of tissue-based lipidomics in forensic investigations of sudden cardiac death, revealing distinct metabolic signatures between hyperacute ischemic heart disease and toxic deaths. Through comprehensive lipidomic profiling of fresh frozen heart tissue, we identified specific lipid signatures that differentiate between these two causes of death. These findings suggest that different mechanisms of cardiac death involve distinct metabolic pathways and cellular responses, potentially offering new biomarkers to help forensic pathologists in an improved diagnosis of death.

## Figures and Tables

**Table 1:** Information from the autopsy.

ID	Sex	Age	Coronary Artery	Histology	Pharmacological Anamnesis	Toxicological Analyses	Cause of Death
1	F	50	Critical stenosis of LCA and AD (85%)	Waviness, contraction band necrosis, fibrosis	None	Negative	Acute ischemic heart disease
2	M	50	Critical stenosis of LCA (80%) and RCA	Fibrosis	None	Negative	Acute ischemic heart disease
3	F	36	Unremarkable	Contraction band necrosis	Methadone	Methadone, benzodiazepines	Drug abuse
4	M	32	Stenosis < 50%	Contraction band necrosis, fibrosis	None	Morphine, cocaine	Drug abuse
5	M	35	Unremarkable	Contraction band necrosis, fibrosis	Levetiracetam, lacosamide	Cocaine, levetiracetam, lacosamide	Drug abuse
6	M	50	Critical stenosis of LCA (90%)	Contraction band necrosis, fibrosis	Levothyroxine	Negative	Acute ischemic heart disease

**Table 2:** PCA loadings for the top 20 PC1 loadings.

	PC1	PC2	PC3	PC4	PC5	PC6
PC 18:1_22:5	0.08268	−0.001322	0.01877	−0.0032998	0.019833	0.0047293
PE 18:1_22:6	0.082675	−0.0018474	0.018954	−0.0038093	0.019489	−0.010676
PC O-36:1_A	0.082265	−0.018953	0.017814	−0.0092145	0.0047633	0.0054953
PC O-36:2_C	0.081399	0.016645	0.00069367	−0.03186	−0.0025386	0.012184
PS 18:0_22:5_B	0.081365	0.016019	0.00068467	−0.032586	−0.0019647	0.02206
PC O-32:0_B	0.081114	0.0095399	0.0253	0.021802	−0.010351	0.016975
DG 18:1/18:1	0.08104	0.0097585	0.025643	0.021807	−0.010522	0.01667
PC O-40:4_A	0.080877	0.014356	0.001999	−0.01839	0.032414	0.0053446
PC 16:0/16:0_A	0.080873	0.014213	0.0017864	−0.018386	0.032557	0.017219
PC 16:0_17:0	0.079403	0.031759	−0.024691	−0.0061435	0.0026323	−0.0042648
PC O-40:5_D	0.0794	0.031783	−0.024647	−0.0064169	0.0023449	0.0034258
SM 34:2;2O	0.079356	0.031872	−0.024789	−0.0065367	0.0023463	0.00348
PC 18:0_18:1_A	0.0787	0.019454	0.0092217	0.01544	0.042007	0.036046
TG 18:1_20:4_22:6	0.078681	0.019355	0.0090687	0.01536	0.042248	0.012975
PE 17:1_18:1	0.078677	0.019372	0.0091107	0.015384	0.042236	0.020715
PI 40:6	0.078587	−0.01689	0.033612	−0.020057	−0.019065	0.020643
LPC 16:0	0.078521	−0.017407	0.033926	−0.019703	−0.01876	0.024681
PI 34:2	0.078172	−0.0078061	0.035193	0.018927	0.027916	−0.0059157
PC O-32:1_B	0.078117	−0.0079559	0.035441	0.019066	0.027765	0.013363
DG O-16:0_16:0	0.078086	−0.0081328	0.035356	0.019247	0.027949	0.0057207

**Table 3:** Most significant lipids according to raw *p*-value and FDR. PC: phosphatidylcholine; SM: sphingomyelin; TG: triacylglycerol (triglyceride); PE: phosphatidylethanolamine; HexCer: hexosylceramide; PI: phosphatidylinositol. Numbers such as 16:0, 18:1, etc., denote fatty acid chain characteristics. The first number indicates the number of carbon atoms in the fatty acid chain (e.g., 16, 18); the number after the colon indicates the number of double bonds (degree of unsaturation) (e.g., 0 = saturated, 1 = one double bond). O- or PC O- indicate an ether-linked lipid species. Symbols such as 3O and 2O represent additional oxygenations or modifications (e.g., hydroxyl groups) on the lipid molecule. Underscores (_) separate fatty acid chains within a lipid (e.g., PC 16:0_18:1 indicates two fatty acid chains, 16:0 and 18:1, in a phosphatidylcholine). Suffixes like _A and _B indicate possible isomeric forms or specific identification codes used in the dataset.

Lipids	t-Stat	*p*-Value	−log10(p)	FDR
PC 16:0_16:2	11.182	0.00036411	3.4388	0.049095
SM 34:1;3O	11.056	0.00038053	3.4196	0.049095
PC O-40:5_C	11.026	0.00038456	3.415	0.049095
SM 40:2;2O_A	5.4796	0.0054	2.2676	0.34317
PC 16:0_18:1_B	5.4733	0.0054225	2.2658	0.34317
TG 16:0_18:1_20:4	5.2722	0.0062027	2.2074	0.34317
PC O-44:6	5.2559	0.0062721	2.2026	0.34317
PC O-34:0_B	−4.3202	0.012446	1.905	0.48536
HexCer 18:1;2O/24:0	−4.3119	0.012527	1.9021	0.48536
PI 38:4	−4.2973	0.012673	1.8971	0.48536
PC 16:0_20:4_B	3.6917	0.020988	1.678	0.67426
PC O-40:8_A	3.6842	0.021125	1.6752	0.67426
PC 18:1_22:4	−3.3692	0.028064	1.5518	0.71071
PE 18:1_20:4_B	−3.3679	0.028098	1.5513	0.71071
PC 17:1_18:1	3.3204	0.029368	1.5321	0.71071
TG 18:0_19:1_22:6	3.3087	0.02969	1.5274	0.71071
PC 17:0_18:0	3.1829	0.033442	1.4757	0.71163
TG 18:0_18:1_22:3	3.174	0.033729	1.472	0.71163
SM 20:1;2O/13:0_A	3.0096	0.039564	1.4027	0.71163
PE 22:1_17:2	−2.9863	0.040484	1.3927	0.71163
PC 18:1_18:2_B	−2.9779	0.040825	1.3891	0.71163
TG O-18:1_22:3_22:5	−2.9766	0.040877	1.3885	0.71163

**Table 4:** Volcano and fold change analyses result.

	FC	log2(FC)	Raw p-Val	−log10(p)
SM 40:2;2O_A	3.5923	1.8449	0.0054	2.2676
PC 16:0_18:1_B	3.5887	1.8434	0.0054225	2.2658
TG 16:0_18:1_20:4	3.8569	1.9474	0.0062027	2.2074
PC O-44:6	3.8589	1.9482	0.0062721	2.2026
PC O-34:0_B	0.32141	−1.6375	0.012446	1.905
HexCer 18:1;2O/24:0	0.3215	−1.6371	0.012527	1.9021
PI 38:4	0.32186	−1.6355	0.012673	1.8971
PC 18:1_22:4	0.42293	−1.2415	0.028064	1.5518
PE 18:1_20:4_B	0.42272	−1.2422	0.028098	1.5513
PC 17:0_18:0	2.1415	1.0986	0.033442	1.4757
TG 18:0_18:1_22:3	2.1372	1.0957	0.033729	1.472
PE 22:1_17:2	0.43626	−1.1967	0.040484	1.3927
PC 18:1_18:2_B	323	−1.6304	0.040825	1.3891
TG O-18:1_22:3_22:5	0.32306	−1.6301	0.040877	1.3885
PC O-44:7	3.3297	1.7354	0.050556	1.2962
PC 16:1_22:6_B	3.327	1.7342	0.050653	1.2954
TG 16:0_18:1_22:6	3.3216	1.7319	0.050772	1.2944
PC 17:0_18:1	2.0392	1.028	0.057225	1.2424
PE 16:0_18:1	2.0349	1.0249	0.057639	1.2393
TG 18:0_18:1_22:4	2.0342	1.0245	0.058514	1.2327
PC 16:1_18:1	2.0255	1.0182	0.058641	1.2318
PC O-42:7	2.0265	1.019	0.058718	1.2312
TG 14:0_20:3_21:1	2.0251	1.018	0.059619	1.2246
Cer 16:1;2O/16:0	2.5321	1.3404	0.07518	1.1239
PE P-18:1_20:1	2.5322	1.3404	0.075568	1.1217
PC 38:5	2.5219	1.3345	0.075859	1.12

**Table 5:** Altered pathways in the toxic group.

Pathway Name	Pathway Lipids	Converted Lipids (Number)	Converted Lipids (Percentage)	Converted Lipids (List)	*p*-Value	Benjamini Correction	Bonferroni Correction
Glycosylphosphatidylinositol (GPI)-anchor biosynthesis	3	2	28.57142857	C00350, C01194	0.000435028	0.003480223	0.006960447
Inositol phosphate metabolism	9	1	14.28571429	C01194	0.112178644	0.163168937	1
Ether lipid metabolism	16	1	14.28571429	C05212	0.191801237	0.236063061	1
Linoleic acid metabolism	25	1	14.28571429	C00157	0.285119368	0.304127326	1
Glycerophospholipid metabolism	26	3	42.85714286	C00157, C00350, C01194	0.003109731	0.012438924	0.049755697
Alpha-linolenic acid metabolism	23	1	14.28571429	C00157	0.265223024	0.303112028	1
Autophagy-other	3	2	28.57142857	C01194, C00350	0.000435028	0.003480223	0.006960447
Arachidonic acid metabolism	75	1	14.28571429	C00157	0.653348996	0.653348996	1
Autophagy-animal	4	2	28.57142857	C00350, C01194	0.000864632	0.004611368	0.013834105
Ferroptosis	11	2	28.57142857	C21480, C21481	0.007585493	0.020227982	0.121367892
Phosphatidylinositol signaling system	11	1	14.28571429	C01194	0.135585354	0180780472	1
Retrograde endocannabinoid signaling	8	2	28.57142857	C00157, C00350	0.003935112	0.012592358	0.062961791
Pathogenic Escherichia coli infection	1	1	14.28571429	C00350	0.013035382	0.029795158	0.208566108
Tuberculosis	5	1	14.28571429	C01194	0.06373131	0.101970097	1
Kaposi’s sarcoma-associated herpesvirus infection	3	1	14.28571429	C00350	0.038669754	0.077339507	0.618716058
Choline metabolism in cancer	5	1	14.28571429	C00157	0.06373131	0.101970097	1

**Table 6:** Pathway analyses in hyperacute case. For each metabolic pathway, “Converted lipids (number)” refers to the number of detected lipid species found to be significantly altered within that pathway based on our statistical criteria. The corresponding percentage is calculated as the ratio of converted lipids to the total number of known lipids in that pathway (per KEGG or LIPEA), multiplied by 100 (e.g., 2 converted out of 7 pathway lipids = 28.6%). To account for multiple hypothesis testing, both the Benjamini–Hochberg method (FDR control) and the more stringent Bonferroni correction were applied, reporting both adjusted *p*-values in the tables. The “Converted lipids (list)” column displays the KEGG compound IDs or standard names of the actual lipids observed to be significantly altered within each pathway. C05212: 1-Radyl-2-acyl-sn-glycero-3-phosphocholine; C00157: 3-sn-Phosphatidylcholine; C00550: Sphingomyelin.

Pathway Name	Pathway Lipids	Converted Lipids (Number)	Converted Lipids (Percentage)	Converted Lipids (List)	*p*-Value	Benjamini Correction	Bonferroni Correction
Glycerophospholipid metabolism	26	1	33.33333333	C00157	0.138577917	0.153975463	1
Ether lipid metabolism	16	1	33.33333333	C05212	0.086905835	0.153975463	0.869058353
Linoleic acid metabolism	25	1	33.33333333	C00157	0.133500773	0.153975463	1
Sphingolipid metabolism	21	1	33.33333333	C00550	0.112992704	0.153975463	1
Arachidonic acid metabolism	75	1	33.33333333	C00157	0.363779183	0.363779183	1
Alpha-linolenic acid metabolism	23	1	33.33333333	C00157	0.123286715	0.153975463	1
Sphingolipid signaling pathway	9	1	33.33333333	C00550	0.049532165	0.123830412	0.495321648
Necroptosis	4	1	33.33333333	C00550	0.022221452	0.123830412	0.222214516
Retrograde endocannabinoid signaling	8	1	33.33333333	C00157	0.044111246	0.123830412	0.441112456
Choline metabolism in cancer	5	1	33.33333333	C00157	0.027724896	0.123830412	0.277248956

## Data Availability

The data supporting the findings of this study are available within the article and its Supplementary Materials. The lipidomic profiles and statistical analyses are included in the Supplementary Materials for further review and analysis.

## Supplementary Materials

The following supporting information can be downloaded at https://www.mdpi.com/article/10.3390/ijms26189031/s1.

## Abbreviations

The following abbreviations are used in this manuscript:

FDR	False Discovery Rate
HexCer	Hexosylceramide
IHD	Ischemic Heart Disease
PC	Phosphatidylcholine
PCA	Principal Component Analysis
PE	Phosphatidylethanolamine
PI	Phosphatidylinositol
PLS-DA	Partial Least Squares Discriminant Analysis
SCD	Sudden Cardiac Death
SVM	Support Vector Machine
SM	Sphingomyelin
UHPLC-Q-TOF	Ultra-High-Performance Liquid Chromatography Quadrupole Time-of-Flight
TG	Triacylglycerol (Triglyceride)
